# External Validation of a Multivariable Diagnostic Prediction Model for Acute Invasive Fungal Rhinosinusitis in Tertiary Care Settings

**DOI:** 10.1002/alr.70024

**Published:** 2025-08-22

**Authors:** Aviv Spillinger, Johanna Ellefson, Qiuyu Yang, Linda X. Yin, Janalee K. Stokken, Thomas Pasic, Ian J. Koszewski, Sandra Y. Lin

**Affiliations:** ^1^ Department of Otolaryngology‐Head & Neck Surgery University of Wisconsin School of Medicine & Public Health Madison Wisconsin USA; ^2^ Department of Surgery University of Wisconsin School of Medicine & Public Health Madison Wisconsin USA; ^3^ Department of Otolaryngology‐Head and Neck Surgery Mayo Clinic Rochester Minnesota USA

**Keywords:** clinical decision rules, invasive fungal infections, sinusitis, validation study

## Abstract

**Background:**

Prompt detection and intervention are crucial for improving outcomes in acute invasive fungal rhinosinusitis (AIFS). Diagnostic prediction models assist in risk‐stratification, but their accuracy requires testing through external validation. This study aims to validate a previously published diagnostic prediction model for AIFS in an independent cohort.

**Methods:**

A retrospective chart review was conducted at a tertiary care center (2008–2023) to identify patients with an otolaryngology consult for suspected AIFS. Of 65 patients identified, 11 (16.9%) were diagnosed with AIFS based on histopathology. Risk was calculated using Yin et al.’s predictive model. Predictive performance was assessed by calibration and discrimination.

**Results:**

Patients had significantly higher rates of diabetes (46.2% vs. 26.1%, *p* = 0.002), long‐term steroid use (60% vs. 28.2%, *p* < 0.0001), and solid organ transplantation (38.5% vs. 8.5%, *p* < 0.001), compared with the development cohort, with conversely lower rates of hematologic malignancy (29.2% vs. 58.7, *p* < 0.001) and neutropenia (19.4% vs. 41%, *p* = 0.001). Despite these differences, both the three‐variable (C‐index: 0.844; 95% CI, 0.736–0.952) and four‐variable models (C‐index: 0.963; 95% CI, 0.919–1) showed adequate discrimination. Both models exhibited slight overprediction of risk, with a calibration‐in‐the‐large predicted risk of 24.1% (95% CI, 13.68–34.46) for the three‐variable model and 24.2% (95% CI, 13.76–34.57) for the four‐variable model. Calibration plots confirmed overprediction.

**Conclusion:**

The AIFS diagnostic model demonstrates acceptable discrimination and calibration on external validation, with generalizability to patients with different comorbidities. Larger studies are recommended to further test the model's predictive performance and clinical applicability.

## 1 Introduction

Diagnostic prediction models are being developed at an accelerating pace across nearly all areas of medicine [1, 2, 3]. Such models estimate the probability of a condition by integrating a number of patient predictors, ranging from demographics to history and examination findings to imaging and laboratory results [4, 5]. Prediction models such as the Fibrosis‐4 Index for liver fibrosis [6] and atherosclerotic cardiovascular disease risk calculators [7] are used to aid clinical decision‐making, such as whether to pursue additional testing or initiate treatment [8].

A previously published diagnostic prediction model for acute invasive fungal sinusitis (AIFS) developed by Yin et al. [9] aimed to guide clinical decision‐making in a disease where early identification and treatment are crucial for improved outcomes, yet nonspecific early signs and symptoms make timely diagnosis challenging [10, 11, 12, 13]. Two related models were developed: a three‐variable model to assist general practitioners in identifying patients with a high enough predicted risk to warrant otolaryngology evaluation, and a four‐variable model to guide otolaryngologists in deciding when to proceed with definitive surgical treatment. The four variables, selected based on their performance in multivariate modeling and their clinical practicality, included: (1) fever ≥38°C; (2) unilateral facial swelling, pain, or erythema; (3) involvement of the orbit or pterygopalatine fossa on CT imaging; and (4) mucosal necrosis on endoscopic examination [9].

While Yin et al.’s [9] AIFS diagnostic prediction model performed well on internal validation, no external validation of this model has been performed to date. External validation involves testing a prediction model on a new cohort of patients to assess its accuracy and generalizability to patients distinct from the development set [3, 14]. Since prediction models generally perform better in the patient cohort from which the model was developed, the performance of internally validated prediction models should be tested and validated in new individuals before their utilization in clinical practice [2, 14]. Afterall, basing clinical decisions on inaccurate prediction models, particularly for serious conditions such as AIFS, can lead to worse patient outcomes [3]. The goal of the present study was to perform an external validation of Yin et al.’s [9] AIFS diagnostic prediction model by evaluating its predictive performance in an independent patient cohort from a separate tertiary care facility.

## 2 Materials and Methods

This study followed published reporting recommendations from the Transparent Reporting of a multivariable prediction model for Individual Prognosis or Diagnosis (TRIPOD) guidelines [8]. No patients or public were involved in the study design. Following institutional review board approval (IRB 2024‐0476), a retrospective chart review was used to identify patients with an otolaryngology consult to evaluate for possible AIFS in a single US tertiary care center from 2008 to 2023. International Classification of Diseases (ICD) 9 and 10 codes for acute sinusitis, orbital cellulitis, and fungal infection were used to screen for AIFS, as done in previous studies [15, 16]. Additional diagnosis codes for hematologic malignancy, solid organ transplant, bone marrow transplant, leukopenia, neutropenia, chronic steroid use, and other immunodeficiency were utilized to identify patients. A positive diagnosis of AIFS was based on formalin‐fixed paraffin‐embedded histopathologic analysis [12, 17].

Retrospective chart review was used to obtain the following data: patient demographics, past medical history, final pathologic diagnosis, and the four predictors of the development prediction model: (1) fever ≥38°C; (2) unilateral facial swelling, pain, or erythema; (3) involvement of the orbit or pterygopalatine fossa on imaging; and (4) mucosal necrosis on endoscopy. Specific definitions of variables and predictors were kept consistent between the development study and the current validation study [9]. Fever was defined as a temperature >38°C within 24 h prior to otolaryngology consultation. Signs of facial swelling and erythema were documented in the physical exam section of the otolaryngology consult notes. Facial pain was identified from self‐reported patient histories. Involvement of the orbit or pterygopalatine fossa was obtained from final radiology reports of CT imaging. Lastly, mucosal necrosis in the nasal cavity was based on endoscopic examination during the initial consultation by an otolaryngology trainee. For patients with multiple consultations to evaluate for AIFS, only data from the initial hospital admission and consultation were abstracted for analysis, such that no duplicates were included. No blinding was performed regarding the status of the AIFS diagnosis.

All baseline characteristics were reported as categorical variables, and Fisher's exact tests were used to compare these between the development and validation cohorts, with *p* < 0.05 representing a significant difference. The predicted risk of AIFS diagnosis for each patient in the external validation study was calculated using the original predictive formula with regression coefficients per predictor and intercept value. Model performance was then assessed by calibration and discrimination. Measures of calibration included calibration‐in‐the‐large and calibration plots. Calibration‐in‐the‐large compares the average predicted risk with the average observed risk for the entire validation cohort, while calibration plots group patients into deciles based on their predicted probabilities [3]. Discrimination was assessed using the concordance index (C‐index), which is equivalent to the area under the curve (AUC) of the receiver operating characteristic (ROC) curves for logistic regression models. A C‐index of 0.5 suggests random concordance, while values of 0.8 and >0.9 represent good and excellent discrimination, respectively [3, 18]. Model performance was evaluated for both the three‐variable model, which excludes an endoscopic exam, and the four‐variable model, which requires an endoscopic exam. No model updating, class imbalance adjustments, or data clustering was performed in the evaluation of model performance. Missing data were removed when calculating the predicted risk of AIFS diagnosis. If an endoscopic exam was missing for an individual, their risk was still calculated for the three‐variable model. Missing values were not imputed. All statistical analyses were performed using R version 4.42.

## 3 Results

A total of 65 patients with a consult indication to evaluate for AIFS were included in the external validation cohort. AIFS was confirmed in 11 cases (16.9%) based on histopathologic analysis. Of the 54 patients without AIFS, 27 (50%) had negative biopsy results. In the remaining cases, the diagnosis was excluded based on low clinical suspicion and/or reassuring findings on nasal endoscopy.

The baseline characteristics and the frequency of predictors for the 65 patients in the validation cohort are summarized in **Table** **1**. Of the 65 patients included in the study, one had missing CT imaging data, and six lacked documentation of a nasal endoscopic examination. These patients with missing data were excluded from calculations of model performance. As a result, 64 patients were included in the evaluation of the three‐variable model (which does not require endoscopic findings), and 58 patients in the evaluation of the full four‐variable model.

**TABLE 1:** Baseline characteristics and predictor frequencies in the validation cohort

Characteristic	Not AIFS (*n* = 54) *n* (%)	AIFS (*n* = 11) *n* (%)
Gender		
Male	35 (65)	8 (73)
Female	19 (35)	3 (27)
Diabetes mellitus	27 (50)	3 (27)
Hyperglycemia (>130 mg/dL)	31 (57)	6 (55)
Hematologic malignancy	16 (30)	3 (27)
Acute myeloid leukemia	7 (13)	3 (27)
Solid organ transplant	21 (39)	4 (36)
Bone marrow transplant	12 (22)	1 (9)
Leukopenia (<4 × 10^9^ cells/L)	21 (39)	3 (27)
Neutropenia (<0.5 × 10^9^ cells/L)	9 (17)	3 (27)
Steroid use		
None	22 (41)	3 (27)
Short term (<30 days)	0 (0)	1 (9)
Long term (>30 days)	32 (59)	7 (64)
**Predictor**		
Fever (≥38°C)		
Yes	15 (28)	5 (45)
No	39 (72)	6 (55)
Missing data	None	None
Unilateral facial swelling, pain, or erythema		
Yes	20 (37)	9 (82)
No	34 (63)	2 (18)
Missing data	None	None
Involvement of the orbit or pterygopalatine fossa on imaging		
Yes	15 (28)	9 (82)
No	38 (70)	2 (18)
Missing data	1 (2)	None
Mucosal necrosis on nasal endoscopy		
Yes	1 (2)	6 (55)
No	49 (91)	3 (27)
Missing data	4 (7)	2 (18)

*Note*: Predictor variables were based on Yin et al.’s diagnostic prediction model and are used to calculate the risk of AIFS diagnosis [9].

Abbreviation: AIFS = acute invasive fungal sinusitis.

A comparison of baseline characteristics between the current validation cohort and the original development cohort is shown in **Table** **2**. Patients in the validation cohort had higher rates of diabetes (46.2% vs. 26.1%, *p* = 0.002), long‐term steroid use (60% vs. 28.2%, *p* < 0.001), and a history of solid organ transplantation (38.5% vs. 8.5%, *p* < 0.001) compared with the development cohort. Conversely, rates of hematologic malignancy (29.2% vs. 58.7%, *p* < 0.001), leukopenia (36.9% vs. 58%, *p* = 0.002), and neutropenia (19.4% vs. 41%, *p* = 0.001) were significantly lower in the validation cohort.

**TABLE 2:** Comparison of baseline characteristics between the validation and development cohorts

Characteristic	Current study (*n* = 65) *n* (%)	Yin et al. [9] (*n* = 283) *n* (%)	*p‐*value
Gender			0.78
Male	43 (66)	181 (64)	
Female	22 (34)	102 (36)	
Diabetes mellitus	30 (46)	74 (26)	**<0.01**
Hyperglycemia (>130 mg/dL)	37 (57)	129 (46)	0.13
Hematologic malignancy	19 (29)	166 (59)	**<0.01**
Acute myeloid leukemia	10 (15)	82 (29)	**0.03**
Solid organ transplant	25 (38)	24 (8)	**<0.01**
Bone marrow transplant	13 (20)	66 (23)	0.63
Leukopenia (<4 × 10^9^ cells/L)	24 (37)	164 (58)	**<0.01**
Neutropenia (<0.5 × 10^9^ cells/L)	12 (18)	116 (41)	**<0.01**
Steroid use			**<0.01**
None	25 (38)	162 (57)	
Short term	1 (2)	41 (14)	
Long term	39 (60)	80 (28)	

**FIGURE 1:**
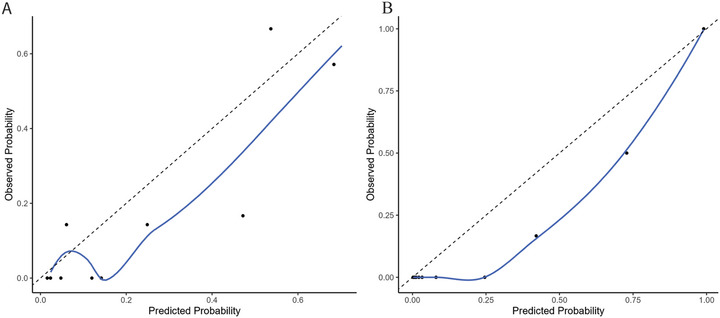
Calibration plots for the (A) three‐variable and (B) four‐variable models. The ten dots represent deciles of the study population, grouped according to their predicted probability of AIFS diagnosis. A smoothed Lowess line is shown in blue.

Eleven of the 65 patients in the validation cohort had pathologic confirmation of AIFS, resulting in an observed risk of 16.9%. Calibration‐in‐the‐large indicated slight overprediction of risk, as the average predicted risk for the entire validation population was 24.1% (95% CI, 13.68–34.46) for the three‐variable model (without endoscopic exam) and 24.2% (95% CI, 13.76–34.57) for the four‐variable model (with endoscopic exam), compared with the observed risk of 16.9%. The calibration plots, which compare predicted risk to observed risk within patient subgroups for both models, are shown in **Figure** **1**. Both the three‐ and four‐variable model calibration plots confirmed overprediction; however, the four‐variable model exhibited near‐perfect calibration at the extremes of predicted risk (i.e., the predicted risk closely mirrored the observed risk for patients with either very high or very low predicted risk).

**FIGURE 2:**
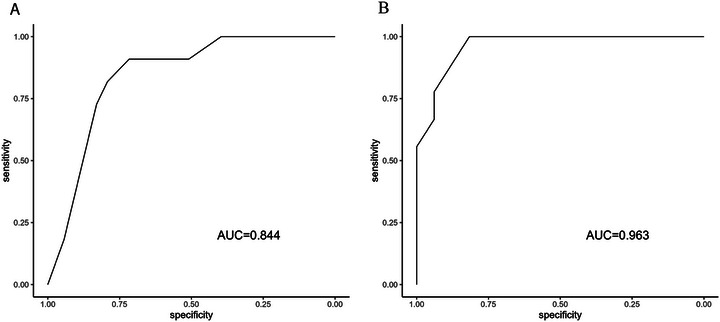
Receiver operating characteristic (ROC) curves for the (A) three‐variable and (B) four‐variable models, used to assess model discrimination. AUC = area under the curve.

Model discrimination, which assesses the model's ability to assign higher predicted risks to patients with AIFS compared with those without, is represented by the AUC of the ROC curves shown in **Figure** **2**. The three‐variable model demonstrated good discrimination, with a C‐index of 0.844 (95% CI, 0.736–0.952). The four‐variable model demonstrated excellent discrimination, with a C‐index of 0.963 (95% CI, 0.919–1). The C‐statistic for the current cohort was nearly identical to the original C‐statistic of the development model (**Table** **3**).

**TABLE 3:** C‐statistic for three‐ and four‐variable models in the development and validation studies

C‐statistic	Current (validation) study	Yin et al. [9]
Three‐variable model	0.844	0.848
Four‐variable model	0.963	0.964

*Note*: The C‐index measures model discrimination. A C‐index of 0.5 indicates random concordance, while values of 0.8 and above suggest good discrimination, with >0.9 representing excellent discrimination.

At a 10% risk cutoff using the three‐variable model, the sensitivity and specificity for predicting AIFS were 90.9% and 50.9%, respectively, while a 75% risk cutoff resulted in a sensitivity of 18.2% and specificity of 94.3%. In comparison, the four‐variable model at a 10% risk cutoff demonstrated a sensitivity of 100% and specificity of 67.4%. Using a 75% risk cutoff with the four‐variable model yielded a sensitivity of 66.7% and specificity of 93.9%.

## 4 Discussion

This study represents the first external validation of a diagnostic prediction model for AIFS. As emphasized in the TRIPOD statement for reporting prediction models and numerous other publications, external validation of diagnostic prediction models is essential prior to their use in real‐world clinical settings [2, 3, 8, 14, 19, 20]. There is currently no standardized diagnostic algorithm for AIFS, a disease with a high mortality rate that requires prompt diagnosis and intervention [21, 22]. Accurate and reliable diagnostic tools that support clinical decision‐making in AIFS are therefore invaluable.

To this end, this validation study demonstrates the adequate predictive performance of Yin et al.’s [9] AIFS diagnostic prediction model when applied to an independent patient cohort. The three‐variable model shows good discrimination with a C‐index of 0.844, while the four‐variable model achieves excellent discrimination with a C‐index of 0.963. The discrimination values are nearly identical to those reported in the original development and internal validation study, despite significant heterogeneity in baseline characteristics between the patients in the validation and development cohorts.

Furthermore, the calibration plots for both models show fairly good calibration, although with a slight trend toward overprediction of risk. Overprediction was further evidenced by the calibration‐in‐the‐large, where predicted risks of 24.1% for the three‐variable model and 24.2% for the four‐variable model exceeded the observed risk of 16.9% in the validation population. While generally undesirable, overprediction of risk may have practical clinical advantages in an AIFS diagnostic tool. Given the rapidly progressive nature of AIFS and the serious morbidity associated with missed or delayed diagnosis, screening models that prioritize sensitivity and exaggerate disease risk can help mitigate the harm of underdiagnosis.

Diagnostic predictive models are statistical tools designed to estimate the probability that an individual patient currently has a particular disease or condition [23]. They are not intended to provide definitive diagnoses, which in the case of AIFS, should be established via gold standard histopathology. Rather, these models are meant to be used alongside sound clinical judgement to support decision‐making regarding the need for further testing or treatment. In their original paper, Yin et al. [9] argue that the diagnostic model should be used as a screening tool in patients with a high pretest probability to identify those who may benefit from otolaryngology consultation or additional, more invasive diagnostic and therapeutic intervention [9].

Notably, adequate performance on external validation was observed despite significant differences in baseline characteristics between the development and validation cohorts, further supporting the model's generalizability. The validation cohort had significantly higher rates of patients with diabetes, long‐term steroid use, and solid organ transplants, whereas the development cohort featured significantly higher rates of patients with hematologic malignancies, neutropenia, and leukopenia. A key component of external validation is demonstrating that a developed model performs well in “similar but different” patients, distinct from the development set [14, 24]. The greater the heterogeneity in population characteristics, the stronger the generalizability of the model [14, 24]. The prediction model validated in the current study does not consider personal attributes such as race, gender, or age. The model integrates clinically practical predictors—subjective history, physical exam findings, CT imaging results, and endoscopic examination—that are routinely obtained during the evaluation of patients for AIFS. While limited evidence exists that Black and Hispanic populations may be disproportionately affected by AIFS, sociodemographic considerations in AIFS remain understudied [16].

Strengths of the current study include adherence to TRIPOD guidelines to the best extent possible. The patient cohort in the study is suitable for external validation and representative of the “at‐risk” target population, as it comprised patients from a different center than the model development cohort and reflects the predominance of AIFS diagnoses in teaching hospitals [19, 25]. Furthermore, AIFS diagnosis was based on “gold standard” permanent section analysis using hematoxylin–eosin staining, increasing the probability that AIFS cases were true positives.

Obvious limitations include the small sample size and the number of positive AIFS cases in the study. It is known that external validation studies require adequate sample sizes to detect relevant declines in model performance, as insufficient sample sizes may result in statistically nonsignificant findings despite the presence of true differences [24, 26]. AIFS is a rare disease, and the present study identified only 11 cases over a 15‐year timeframe. Additionally, missed cases are possible, as patient identification relied on ICD codes, which are prone to misclassification and miscoding; no specific ICD code exists for AIFS. Finally, although both the development and validation studies were conducted at academic centers within geographically similar regions, differences in patient comorbidities between the two cohorts suggest that the populations were sufficiently distinct to support meaningful conclusions about the model's generalizability.

To reach sufficient sample sizes and improve confidence in the predictive performance of the AIFS diagnostic model, larger, multicenter studies are recommended. Including centers from diverse settings with varying case‐mixes will also enhance the model's generalizability. These larger datasets may also enable model updating, which was not attempted in the present study due to adequate results observed on external validation. Examples of model updating can include the incorporation of MRI results, which can add additional diagnostic value due to their ability to evaluate enhancement characteristics of the sinonasal mucosa [21, 27, 28, 29].

## 5 Conclusion

There is a pressing need for accurate diagnostic prediction models to guide clinical decision‐making in AIFS, given the complex balance between avoiding misdiagnosis with the need to reduce costs and morbidity associated with invasive diagnostics and intervention. This study presents the first external validation of an AIFS diagnostic prediction model integrating clinically practical predictors commonly obtained during AIFS evaluation. The model demonstrates adequate predictive performance, with acceptable model discrimination and calibration, though a slight trend toward overprediction of risk. Larger, multicenter studies are recommended to further evaluate the model's predictive performance and strengthen confidence in its use in clinical practice.

## Conflicts of Interest

The authors declare no conflicts of interest.
